# Editorial: Rhizosphere interactions: root exudates and the rhizosphere microbiome

**DOI:** 10.3389/fpls.2023.1281010

**Published:** 2023-09-05

**Authors:** Linkun Wu, Leslie A. Weston, Shusheng Zhu, Xingang Zhou

**Affiliations:** ^1^ College of Life Sciences, Fujian Agriculture and Forestry University, Fuzhou, Fujian, China; ^2^ Gulbali Institute for Agriculture, Water and the Environment, Charles Sturt University, Wagga Wagga, NSW, Australia; ^3^ State Key Laboratory for Conservation and Utilization of Bio-Resources in Yunnan, College of Plant Protection, Yunnan Agricultural University, Kunming, China; ^4^ College of Horticulture, Northeast Agricultural University, Xiangfang, Harbin, China

**Keywords:** root exudates, rhizosphere microbial community, rhizosphere cross-talk, plant-microbe interactions, plant health

The rhizosphere, a term introduced by Lorenz Hiltner in 1904, is defined as a thin layer of soil that surrounds and is influenced by plant roots (Philippot et al., 2013). It can be divided into several distinct zones, including the endorhizosphere, the rhizoplane, and the ectorhizosphere (Morgan et al., 2005). The rhizosphere is one of the key interfaces between plants and their environment, with intensive root-induced physical, chemical, and biological processes (Zhang et al., 2022). It is also considered a hotspot for plant-microbe interactions because plant roots release enormous amounts of photosynthetically fixed carbon into the surrounding soil. Root exudation typically creates a nutrient-rich rhizosphere microenvironment in which microbial activity is stimulated. Root exudates consist of a wide variety of primary and secondary compounds, including carbohydrates, amino acids, and organic acids, phenolics, flavonoids, auxins (Zhu et al., 2016). They provide a readily bioavailable supply of nutrients and energy for microbial growth and also act as a signaling messenger to shape the rhizosphere microbiome (Luo et al., 2020; Koprivova and Kopriva, 2022). The rhizosphere microbiome, referred to as the plant’s second genome, plays a crucial role in plant growth and health (Berendsen et al., 2012). The rhizosphere is colonized by a huge number of microorganisms and invertebrates, which exert either positive, negative, or neutral effects on plant growth and fitness. In recent years, there has been a growing interest in exploring plant-microbe rhizosphere interactions, termed rhizosphere cross-talk, in natural and agricultural ecosystems.

The number of records retrieved through Web of Science with keywords ‘rhizosphere interactions’ or ‘plant-microbe interactions’ or ‘rhizosphere crosstalk’ or ‘plant–soil feedbacks’ or ‘rhizosphere microbiome’ increased from 406 (1948–2000) to 1924 (2001-2010) and then to 11,451 (2011-2023) (data retrieved August 12, 2023). As a response to the importance of plant-microbiome rhizosphere interactions, we proposed the Research Topic “*Rhizosphere Interactions: Root Exudates and Rhizosphere Microbiome*”. This Research Topic aims to present current information on trends and methods utilized in the study of plant-microbe rhizosphere interactions and also highlights the multifaceted research approaches used to characterize root exudates, the associated rhizosphere microbiome, and interactions among the two. In this Research Topic, we have collected 22 original research articles that contribute to expanding our knowledge about the mechanisms of plant-microbe interactions and their significance for plant growth and soil health, the key factors framing the microbial community in the rhizosphere, and the applications of rhizosphere interactions for sustainable agriculture, forest, and environmental management.

## Rhizosphere microbiome and plant health

Plants and their microorganisms have established intimate associations throughout the length of their evolutionary history. It is increasingly recognized that plants could be considered as ‘meta-organisms’ or ‘holobionts’, consisting of the plant itself plus the associated microbiota (Snelders et al., 2022). The rhizosphere microbiome can directly or indirectly influence plant growth, development, and health by modulating plant nutrient uptake and/or resistance to abiotic and biotic stress. Wang H. et al. indicate that the rhizosphere microbiome could regulate plant drought tolerance of *Atractylodes lancea*. Zhang J. et al. reveal the close relationships between the rhizosphere microbial community and corm rot disease resistance of *Crocus sativus*.

Among the beneficial microbes, plant growth-promoting rhizobacteria (PGPR) and fungi (PGPF) can facilitate a host’s growth and health through various mechanisms, including improving soil structure and nutrient availability, modulating/producing plant hormones, and preventing phytopathogens by direct antibiosis or inducing systemic resistance. For instance, the enrichment of PGPR, mediated by intercropping with maize, could significantly promote the growth of *A. lancea* (Peng Z. et al.). Zhang F. et al. demonstrate that PGPR members (i.e. *Bacillus Megaterium*) of the genus *Bacillus* benefit the competitive growth and successful invasion of *Ambrosia artemisiifolia* by increasing available nutrient levels. Recently, there has been an increasing interest in utilizing PGPRs for the biocontrol of soil-borne diseases (Wu et al., 2020; El-Saadony et al., 2022). Pu et al. indicate that pre-inoculation of arbuscular mycorrhizal fungi enhances disease resistance of *Salvia miltiorrhiza* to *Fusarium* wilt by inducing the expression of defense enzymes and defense-related genes. Han et al. find that the *Pseudomonas* strain ZL8 isolated from the sclerotium of *Polyporus umbellatus* exhibits broad-spectrum antifungal activity and could promote the growth of *Salvia miltiorrhiza* by inhibiting its wilting. In addition, it should be noted that the rhizosphere microbiome is reported to be vital for the accumulation of active ingredients and the quality formation of medicinal plants (Köberl et al., 2013). Chen J. et al. provide insights into relationships between root metabolism and rhizosphere microbiota of *Angelica sinensis* at different growth stages. Wang J. et al. find that the application of *Burkholderia ambifaria* LK-P4 could promote the growth and the content of specific active ingredients in *Anoectochilus roxburghii*.

## Rhizosphere microbiome assembly and its driving factors

Given the importance of the rhizosphere microbiome for plant growth, an in-depth understanding of rhizosphere microbiome assembly and its driving factors is key to rhizosphere engineering for sustainable crop production. The composition and structure of the rhizosphere microbial community can be influenced by many factors, including plant domestication, plant genotype, plant development stage, plant compartment, root exudates, soil type, growth conditions, and agricultural practice (Edwards et al., 2015; Chen et al., 2019; Qu et al., 2020; Bai et al., 2022; Guo et al., 2022; Luo et al., 2022). For instance, Peng B. et al. reveal the strong effects of drip irrigation on the rhizosphere bacterial community of cotton as compared with traditional flood irrigation, and indicate that drip irrigation under plastic film mulch alters the core bacterial network module and suppresses soil nutrient cycling. Huang et al. indicate that on- and off-year management practices affect soil organic carbon sequestration by changing soil microbial communities of *Phyllostachys edulis*. Among the aforementioned driving factors, soil physicochemical properties including soil pH could directly or indirectly affect the structure and function of rhizosphere microbiota (Wei et al., 2018; Xun et al., 2019; Zhang et al., 2022). Lin et al. indicate that soil acidification results in significant changes in soil microbial community structure and the abundance of genes involved in the soil nitrogen cycle. Similarly, Lu et al. find that the excessive application of K_2_SO_4_ fertilizer increases soil acidification and alters the rhizosphere microbial community and functions. Recently it has been reported that cropping patterns such as monoculture, intercropping, and crop rotation exerted a strong effect on the rhizosphere microbiome assembly (Wu et al., 2020; Li et al., 2022; Zhou et al., 2023). For example, consecutive monoculture of blueberry, Chinese fir, and tobacco remarkably alter the assembly of microbial communities in the rhizosphere (Che et al.; Chen J. et al.; Wang P. et al.).

Root exudates, acting as substrates and signaling molecules for microbes, are another critical factor modulating the assembly of the rhizosphere microbiome (Chagas et al., 2018; Bai et al., 2022). On the one hand, root exudates are known to have specialized roles in plant–plant communication (Kong et al., 2018). Li J. et al. indicate that allelochemicals including benzoic acid and cinnamic acid derivatives secreted by allelopathic rice roots play important roles in inhibiting surrounding weeds. On the other hand, root exudates are the key regulators in plant-microbe cross-talk, and can modify both biological and physical interactions between roots and soil microorganisms by mediating various positive and negative plant-microbe interactions (Berendsen et al., 2012; Deng et al., 2023). Root exudates, particularly those containing specific secondary metabolites, play crucial roles in shaping the rhizosphere microbiome by recruiting or repelling different community members (Voges et al., 2018; Li et al., 2023). Sun et al. demonstrate that the root exudates of *Flaveria bidentis* could significantly increase the abundances of *Bacillus frigoritolerans* and *Bacillus megaterium* and promote their nitrogen-fixing and phosphate-solubilizing abilities, which further increases soil available phosphorus and nitrogen levels and promotes the invasiveness of *F. bidentis*. Li Q. et al. show that the dominant chemoattractants (i.e. 2,4-di-tert-butylphenol, methyl stearate, and arginine) in the root exudates mediate the rhizosphere bacterial community assembly of *Casuarina equisetifolia* L.

Increasing evidence has shown that plant allelopathy, replant disease, and interspecific facilitation in intercropping systems are facilitated by the integrative effects of plant-microbe interactions mediated by root exudates (Li et al., 2020; Mwendwa et al., 2021; Xu et al., 2021; Zhou et al., 2023). Yang et al. indicate that root exudates of *Rehmannia glutinosa* could stimulate the proliferation of *Fusarium oxysporum*, which alters the expression patterns of Leucine-rich repeat receptor-like protein kinases (*RgLRRs*), disorders the growth and development of *R. glutinosa*, and finally results in the formation of replant disease. However, intercropping with *Achyranthes bidentata* alleviates *Rehmannia glutinosa* replant disease by modulating root exudates and improving the rhizosphere microenvironment (Liu et al.). Furthermore, An et al. indicate that alfalfa cultivars affect rhizosphere microbial biomass and community composition. Li Q. et al. investigate the bacterial and fungal communities in multiple compartment niches of *Casuarina equisetifolia* L., and find that ecological niche selection shapes the assembly and diversity of microbial communities. Ultimately, the effects of plant domestication, plant genotype, plant development stage, and plant compartment on the assembly of rhizosphere microbiomes have also been reported to be associated with the changes in root exudation profile (Zhalnina et al., 2018; Oyserman et al., 2022; Yue et al., 2023). Therefore, a deeper understanding of the spatiotemporal dynamics of root exudates is vital in disentangling the chemical communication between plants and microbes and modulating the rhizosphere microbiome for plant fitness and sustainable agriculture.

## Outlook and future challenges

Plants have evolved over millions of years, with surrounding microbiota including mutualists, pathogens, and commensals, through diverse signaling mechanisms. Despite great progress in understanding the assembly and functions of the rhizosphere microbiome, a huge gap still exists in the understanding of the complex mechanisms of plant–microbe cross-talk in the rhizosphere and the application of beneficial microbiomes for sustainable agriculture, horticulture, and forestry. More recently, remarkable progress related to the methods and technologies used when performing rhizosphere ecological research has been noted. For example, the use of metabolomics coupled with imaging technology has resulted in key information on the localization of the production and release of secondary plant products. Stable isotope probing (SIP) of DNA and RNA combined with high throughput sequencing has enabled the characterization of the active rhizosphere microbiome that utilizes root exudates for nutrient and energy requirements. Furthermore, the use of synthetic microbial communities (SynComs) has enriched our knowledge of plant-microbe and microbe-microbe interactions as well (Liu et al., 2019). It is optimistically recognized that a deeper and more fine-grained understanding of the precise mechanisms underlying the rhizosphere interactions between plants and other organisms will be achieved in the near future, thanks to advanced techniques such as high-throughput sequencing technologies, integrating omics approaches, system molecular biology, non-invasive *in situ* analyses, and high-performance computing.

To date, the construction and application of beneficial microbial consortia in agriculture production continue to present a great challenges. Many biocontrol agents are effective under experimental conditions but perform poorly in complex field environments, which is mainly attributed to poor rhizosphere colonization and persistence (Bai et al., 2022; Yang et al., 2023). Therefore, one future direction is to explore the environmental factors and microbial phenotypes required for colonization and persistence in the rhizosphere environment. In addition, it is necessary to disentangle the genetic basis of rhizosphere microbiome assembly and identify plant genes that regulate microbial colonization. Breeding for improved cultivars with beneficial microbial interactions is a potentially effective way to engineer the rhizosphere microbiome and promote plant growth and fitness. Overall, novel discernment of the biotic and abiotic factors that shape the rhizosphere microbiome will be crucial in harnessing the most beneficial microbiome to enhance agricultural productivity and ecosystem functions.

## Author contributions

LW: Conceptualization, Writing – original draft, Writing – review & editing, Formal Analysis. LAW: Formal Analysis, Writing – review & editing. SZ: Formal Analysis, Writing – review & editing. XZ: Formal Analysis, Writing – review & editing.
